# Immunohistochemical Analysis of PD-L1 Expression in Canine Malignant Cancers and PD-1 Expression on Lymphocytes in Canine Oral Melanoma

**DOI:** 10.1371/journal.pone.0157176

**Published:** 2016-06-08

**Authors:** Naoya Maekawa, Satoru Konnai, Tomohiro Okagawa, Asami Nishimori, Ryoyo Ikebuchi, Yusuke Izumi, Satoshi Takagi, Yumiko Kagawa, Chie Nakajima, Yasuhiko Suzuki, Yukinari Kato, Shiro Murata, Kazuhiko Ohashi

**Affiliations:** 1 Department of Disease Control, Graduate School of Veterinary Medicine, Hokkaido University, Sapporo, Japan; 2 Veterinary Teaching Hospital, Graduate School of Veterinary Medicine, Hokkaido University, Sapporo, Japan; 3 North Lab, Sapporo, Japan; 4 Department of Diagnostic Pathology, Graduate School of Veterinary Medicine, Hokkaido University, Sapporo, Japan; 5 Research Center for Zoonosis Control, Hokkaido University, Sapporo, Japan; 6 Department of Regional Innovation, Graduate School of Medicine, Tohoku University, Sendai, Japan; Mie University Graduate School of Medicine, JAPAN

## Abstract

Spontaneous cancers are common diseases in dogs. Among these, some malignant cancers such as oral melanoma, osteosarcoma, hemangiosarcoma, and mast cell tumor are often recognized as clinical problems because, despite their high frequencies, current treatments for these cancers may not always achieve satisfying outcomes. The absence of effective systemic therapies against these cancers leads researchers to investigate novel therapeutic modalities, including immunotherapy. Programmed death 1 (PD-1) is a costimulatory receptor with immunosuppressive function. When it binds its ligands, PD-ligand 1 (PD-L1) or PD-L2, PD-1 on T cells negatively regulates activating signals from the T cell receptor, resulting in the inhibition of the effector function of cytotoxic T lymphocytes. Aberrant PD-L1 expression has been reported in many human cancers and is considered an immune escape mechanism for cancers. In clinical trials, anti-PD-1 or anti-PD-L1 antibodies induced tumor regression for several malignancies, including advanced melanoma, non-small cell lung carcinoma, and renal cell carcinoma. In this study, to assess the potential of the PD-1/PD-L1 axis as a novel therapeutic target for canine cancer immunotherapy, immunohistochemical analysis of PD-L1 expression in various malignant cancers of dogs was performed. Here, we show that dog oral melanoma, osteosarcoma, hemangiosarcoma, mast cell tumor, mammary adenocarcinoma, and prostate adenocarcinoma expressed PD-L1, whereas some other types of cancer did not. In addition, PD-1 was highly expressed on tumor-infiltrating lymphocytes obtained from oral melanoma, showing that lymphocytes in this cancer type might have been functionally exhausted. These results strongly encourage the clinical application of PD-1/PD-L1 inhibitors as novel therapeutic agents against these cancers in dogs.

## Introduction

Dogs often develop spontaneous cancers, which may cause death [1] or reduce the quality of life of the patients. Among malignant cancers, lymphoma, mast cell tumor (MCT), osteosarcoma (OS), soft tissue sarcoma, and mammary carcinoma are relatively frequent [2,3] and often recognized as fatal diseases in clinical settings. Oral melanoma and hemangiosarcoma (HSA) are also common malignancies in dogs, and patient dogs with these types of cancer usually have poor prognosis [4,5]. The standards of care for each cancer type have been established and successfully improved the prognosis; however, the treatment outcomes are not always satisfying because of the low-to-moderate response rates or limited prolongation of survival times.

Immunotherapies have potential as novel treatment options for cancer. Among those, immune checkpoint inhibitors such as anti-programmed death 1 (PD-1) and anti-PD-ligand 1 (PD-L1) antibodies showed promising effects on several malignancies in humans [6,7]. The immune checkpoint molecule PD-1 is a CD28-family receptor, which suppresses immune responses. PD-1 on T cells negatively regulates T cell receptor signaling and inhibits the induction of cytokines such as interferon gamma (IFN-γ), interleukin 2, and tumor necrosis factor alpha as well as cell proliferation, allowing the maintenance of peripheral tolerance or the persistence of certain pathogens in the host [8]. Two ligands for PD-1 have been reported: PD-ligand 1 (PD-L1) and PD-L2. PD-L2 expression is restricted to certain types of cells or tissues, such as macrophages and dendritic cells, whereas PD-L1 expression can be induced in a wide variety of cell types, including non-hematopoietic cells. PD-L1 expression is not found in most normal tissues; however, its expression is reported in various tumor cells [9,10] and thus is considered as one of the immune evasion mechanisms for cancer. In renal cell carcinoma, gastric cancer, and other cancer patients, PD-L1 expression is known to be associated with poor prognosis [10–12], indicating that PD-L1 could be an important regulator of the immune system when it fights against cancer. Antibody drugs that target this pathway interfere with binding of PD-L1 to PD-1 and can enhance specific immune responses to tumor cells [13,14], subsequently resulting in the regression of cancer [15]. A number of clinical trials have revealed that objective responses can be obtained with anti-PD-1 or anti-PD-L1 antibodies in patients with malignant cancers, including advanced melanoma, non-small cell lung carcinoma, and renal cell carcinoma [6,7], and accumulating evidence demonstrates the potential of immune checkpoint inhibitors in cancer treatment.

However, there are only few reports on the PD-1/PD-L1 axis in dogs, and its association with diseases remains to be elucidated. Previously, we have reported that PD-L1 is expressed in dog melanoma, MCT, and renal cell carcinoma and that PD-L1 blockade by an anti-PD-L1 antibody enhances IFN-γ production by tumor-infiltrating cells [16]. These results suggest that anti-PD-L1 antibodies may have therapeutic effects on cancers in dogs. In this study, to assess which cancer types could be targeted, PD-L1 expression in dog malignant cancers was further investigated. Here, we show that dog oral melanoma, OS, HSA, MCT, mammary adenocarcinoma, and prostate adenocarcinoma express PD-L1, suggesting that the PD-1/PD-L1 axis could be used as an immune evasion mechanism in these cancers. In support of this hypothesis, PD-1 expression was upregulated on tumor-infiltrating lymphocytes obtained from oral melanoma and hepatic tumors. At least, therapeutic potentials of PD-1/PD-L1 blockers in oral melanoma and other PD-L1-positive cancers deserve further investigation.

## Materials and Methods

### Canine samples

Animal use throughout this study was approved by the Institutional Animal Care and Use Committee (the serial number of approval was #15–0149), Hokkaido University. No animal was sacrificed for the purpose of this study. Clinical samples of dog tumor tissues were surgically excised at the Veterinary Teaching Hospital, Graduate School of Veterinary Medicine, Hokkaido University and at Veterinary Hospitals in Sapporo city in 2014–2015. Upon the use for this study, informed consent was obtained from all owners of the dogs. Peripheral blood samples of clinically healthy dogs were collected from beagles at the age of 12–15 years kept at the Experimental Animal Facility, Graduate School of Veterinary Medicine, Hokkaido University.

### Flow cytometry

To assess the binding specificity of a recently established anti-PD-L1 monoclonal antibody (mAb) [17], flow cytometric analyses were performed as described previously [16], with some modifications. In brief, canine melanoma cell lines, CMeC and CMM-1, which are PD-L1-negative under normal conditions [16], were transfected with the pEGFP-N2 vector or the pEGFP-N2–cPD-L1 vector [16] using Lipofectamine 2000 (Life Technologies, Carlsbad, CA, USA) according to the manufacturer’s instructions. One day after the transfection, the cells were harvested and stained with anti-PD-L1 mAb 6G7-E1 or an isotype-matched control antibody (rat IgM) (BD Biosciences, San Jose, CA, USA) at a final concentration of 10 μg/mL. After washing, the cells were incubated with an allophycocyanin-conjugated anti-rat Ig antibody (Beckman Coulter, Fullerton, CA, USA). Fluorescence was analyzed by a FACSVerse flow cytometer (BD Biosciences) and the FCS Express 4 software (De Novo Software, Glendale, CA, USA). For transfected cells, enhanced green fluorescent protein (EGFP)-positive cells were gated and used for analysis.

To assess whether PD-1 expression is upregulated in the context of canine cancers, flow cytometric analysis was performed with cross-reactive anti-human PD-1 antibody [18]. Peripheral blood mononuclear cells (PBMCs) and TILs from freshly excised dog tumor tissues were obtained as described previously [16]. All single cell suspensions were prepared by the mechanical method. Cells were stained with the optimal concentrations of FITC-conjugated anti-CD4/PE-conjugated anti-CD8 antibody cocktail (AbD Serotec, Oxford, UK) and biotinylated anti-human PD-1 antibody (R&D Systems, Minneapolis, MN, USA) for 30 min at room temperature (RT). After washing, cells were incubated with Streptavidin APC-eFluor 780 (eBioscience, San Diego, CA, USA) for 30 min at RT. Fluorescence of the cells were analyzed by FACS Verse and FACS Express 4 software. Biotinylated normal goat IgG (R&D systems) was used as an isotype-matched control antibody. For the analysis, lymphocytes population was gated by FSC and SSC and within them PD-1 expression on CD4^+^ cells or CD8^+^ cells was evaluated.

### Specimens

Specimens were randomly selected from formalin-fixed and paraffin-embedded tissues of dog malignant tumors (110 samples in total), which had been submitted for histological diagnosis and kept in a commercial pathology laboratory (North Lab, Hokkaido, Japan). All the melanoma samples (*n* = 40) used in this study originated from the oral cavity. The MCT samples (*n* = 5) were all classified into Patnaik grade III [19]. For mammary adenocarcinoma sample (*n* = 5) analysis, no inflammatory mammary carcinoma samples were included. In addition to the above-mentioned specimens, OS (*n* = 10), HSA (*n* = 10), prostate adenocarcinoma (*n* = 5), skin squamous cell carcinoma (*n* = 5), diffuse large B-cell lymphoma (*n* = 5), nasal adenocarcinoma (*n* = 5), soft tissue sarcoma (*n* = 5), histiocytic sarcoma (*n* = 5), transitional cell carcinoma (*n* = 5), and anal sac gland carcinoma with metastatic disease (*n* = 5) specimens were studied by immunohistochemistry.

### Immunohistochemistry

Immunohistochemical staining was examined by the avidin–biotin peroxidase complex (ABC) procedure. Sections (4 μm) were dewaxed in xylene and hydrated through graded alcohols. To remove endogenous peroxidase, the sections were immersed in a 3% hydrogen peroxide solution at room temperature for 10 min. Following this, the sections were incubated with anti-PD-L1 antibody 6G7-E1 (10 μg/mL). After washing with phosphate-buffered saline, the sections were incubated in a secondary antibody solution (biotin-labeled goat anti-rat IgM) (Jackson ImmnunoResearch Laboratories, West Grove, PA, USA) at room temperature for 30 min. Following the incubation, the sections were reacted using the ABC in the VECTASTAIN Elite ABC Kit (Vector Laboratories, Burlingame, CA, USA) at room temperature for 30 min. Visualization was accomplished using a 0.05% 3,3′-diaminobenzidine solution. Mayer’s hematoxylin stain was used as a counterstain. For the negative control, sections were subjected to the same procedures without the primary antibody or with the rat IgM isotype control antibody (Acris Antibodies, Herford, Germany). The specimen was considered positive for PD-L1 if there was histologic evidence of cell staining. No obvious staining was observed in the negative control sections (S1 Fig). To prepare the OS specimens, decalcification was performed using a standard protocol with Plank–Rychlo’s solution. Berlin blue staining was performed to distinguish hemosiderin deposits from positive staining in the HSA samples, when required.

### Statistical analysis

In the flow cytometric analysis of PD-1 expression, Mann–Whitney U test was conducted and *p* < 0.05 was considered to be statistically significant.

## Results

### Anti-PD-L1 antibody 6G7-E1 specifically binds to dog PD-L1

**Fig 1:**
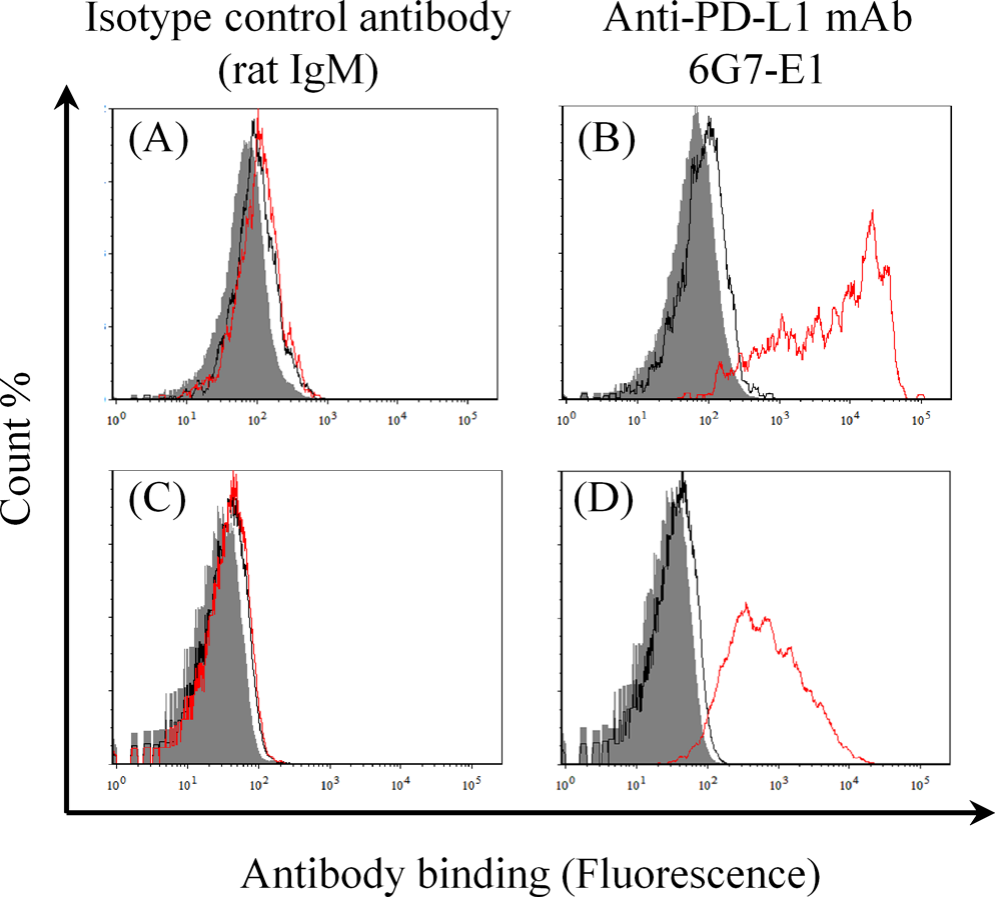
Binding specificity of anti-PD-L1 mAb 6G7-E1. Canine melanoma cell lines, CMeC and CMM-1, were transfected with the pEGFP-N2 vector (mock) or pEGFP-N2–cPD-L1, and antibody binding was analyzed by a flow cytometer. The shaded area, black line, and red line represent the results for the cells with no treatment, mock-transfected cells, and cPD-L1–EGFP-transfected cells, respectively. (A) Binding of the isotype control antibody to CMeC; (B) binding of 6G7-E1 to CMeC; (C) binding of the isotype control antibody to CMM-1; and (D) binding of 6G7-E1 to CMM-1 are shown in the histograms. No binding of 6G7-E1 was observed in the mock-transfected or untransfected cells, whereas specific binding was observed in the cPD-L1–EGFP-transfected cells.

The anti-PD-L1 antibodies that can bind to dog PD-L1 were established previously [16,17]. Among those, 6G7-E1 showed sufficient binding as determined by immunohistochemistry of formalin-fixed and paraffin-embedded dog tumor sections. To test the specificity of 6G7-E1 in detection of dog PD-L1, we first tried Western blotting analysis of canine PD-L1–rabbit IgG Fc fusion protein [16] as a positive control. However, no binding was observed when 6G7-E1 was used as the primary antibody, while an anti-rabbit IgG Fc antibody detected the specific band (data not shown). Because this result suggests that 6G7-E1 recognizes a specific conformation of dog PD-L1, flow cytometric analysis of the canine melanoma cell lines CMeC and CMM-1 was performed. No binding was observed with the non-transfected or mock (EGFP only)-transfected canine melanoma cells, whereas 6G7-E1 was bound to the cells transfected with canine PD-L1–EGFP (Fig 1), indicating specific binding of 6G7-E1 to dog PD-L1.

### Canine malignant cancers express PD-L1

**Fig 2:**
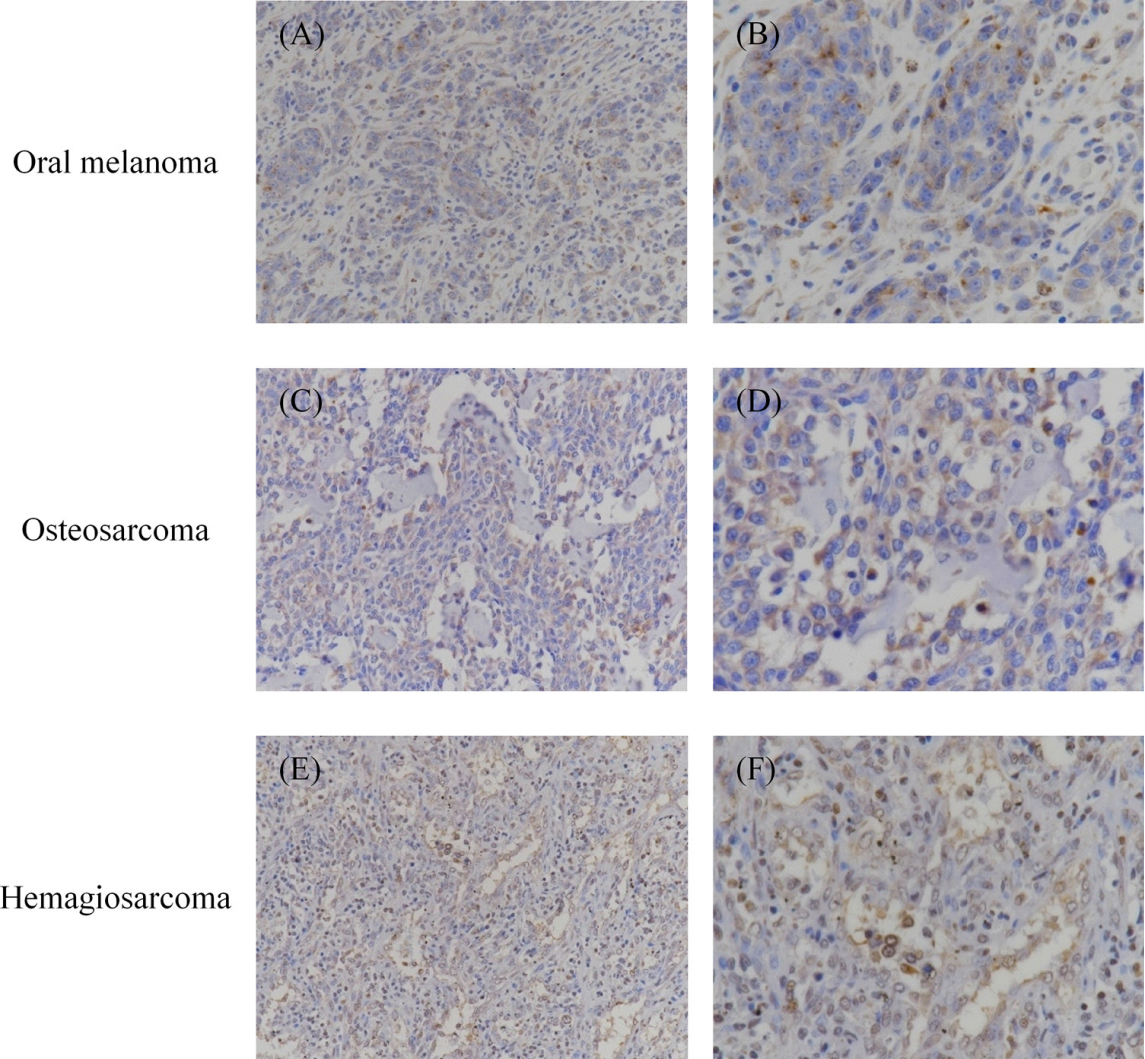
Immunohistochemical analysis of PD-L1 in oral melanoma, osteosarcoma, and hemangiosarcoma. Formalin-fixed and paraffin-embedded tumor tissues were examined immunohistochemically. The sections were stained with anti-PD-L1 mAb 6G7-E1. Representative positive stainings of (A, B) oral melanoma, (C, D) osteosarcoma, and (E, F) hemangiosarcoma are shown. Original magnification: (A, C, E) 200×; (B, D, F) 400×.

**Fig 3:**
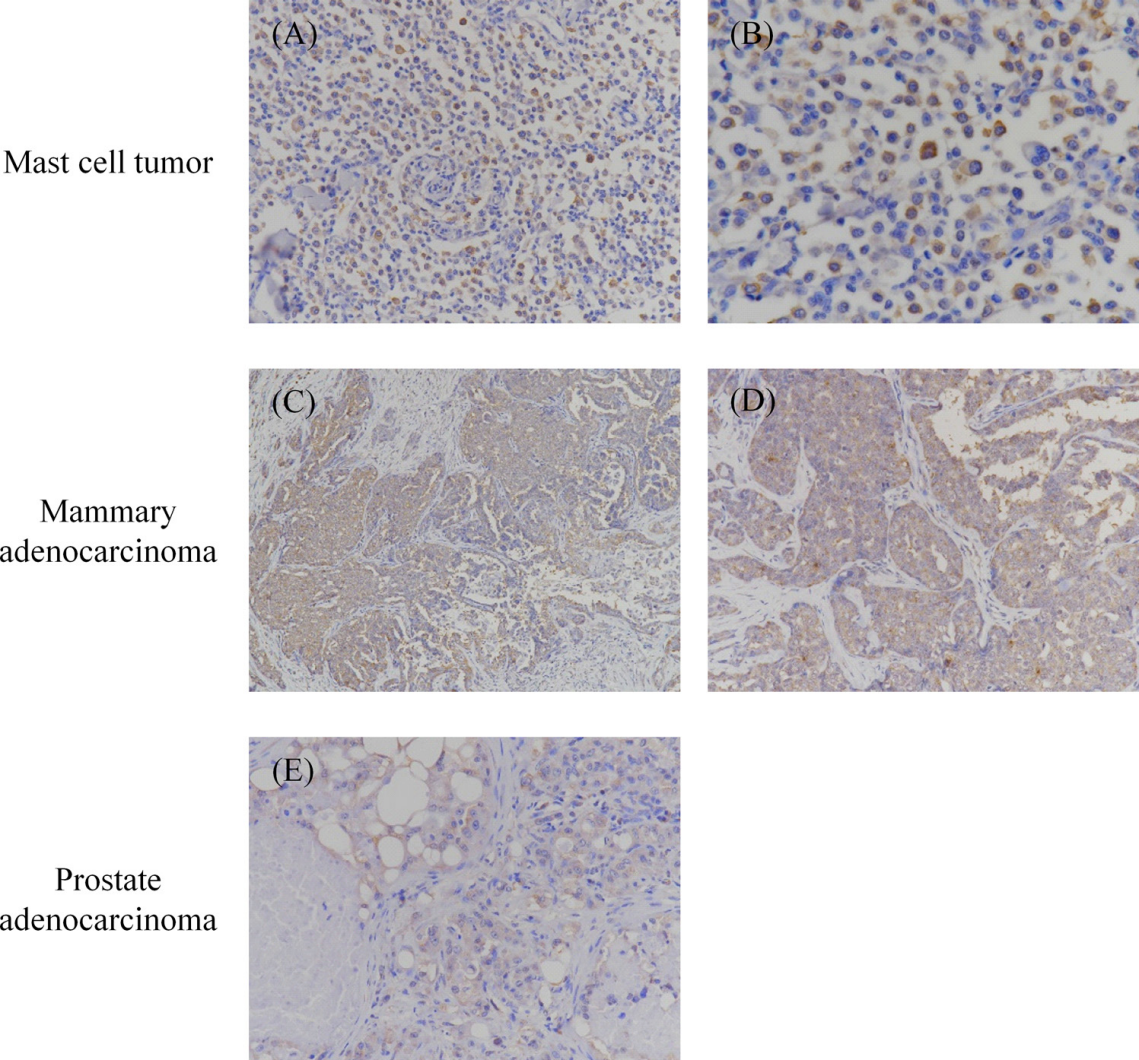
Immunohistochemical analysis of PD-L1 in mast cell tumor, mammary adenocarcinoma, and prostate adenocarcinoma. Formalin-fixed and paraffin-embedded tumor tissues were examined immunohistochemically. The sections were stained with anti-PD-L1 mAb 6G7-E1. Representative positive stainings of (A, B) mast cell tumor, (C, D) mammary adenocarcinoma, and (E, F) prostate adenocarcinoma are shown. Original magnification: (A, D, E) 200×; (B) 400×; (C) 100×.

Immunohistochemical analysis was performed to detect PD-L1 expression in various malignant cancers. In total, 110 specimens were tested, among which 36 oral melanoma, 7 OS, 6 HSA, 3 grade III MCT, 4 mammary adenocarcinoma, and 3 prostate adenocarcinoma specimens were PD-L1 positive (Figs 2 and 3, and Table 1). Both cytoplasmic staining and cell-surface staining were observed. The rates of PD-L1 expression were 90% (36/40) for oral melanoma, 70% (7/10) for OS, 60% (6/10) for HSA, 60% (3/5) for grade III MCT, 80% (4/5) for mammary adenocarcinoma, and 60% (3/5) for prostate adenocarcinoma (Table 1). The other 7 types of tumors, namely squamous cell carcinoma, diffuse large B-cell lymphoma, nasal adenocarcinoma, soft tissue sarcoma, histiocytic sarcoma, transitional cell carcinoma, and anal sac gland carcinoma did not express PD-L1, although the tested sample size was substantially limited (5 cases for each tumor type).

**Table 1:** PD-L1 expression in various canine malignant cancers.

Pathology	Positive cases/Tested samples
Melanoma (oral)	36/40
Osteosarcoma	7/10
Hemangiosarcoma	6/10
Mast cell tumor (grade III)*	3/5
Mammary adenocarcinoma**	4/5
Prostate adenocarcinoma	3/5
Squamous cell carcinoma (skin)	0/5
Diffuse large B-cell lymphoma	0/5
Nasal adenocarcinoma	0/5
Soft tissue sarcoma	0/5
Histiocytic sarcoma	0/5
Transitional cell carcinoma	0/5
Anal sac gland carcinoma	0/5

The results of immunohistochemical analysis were summarized.

*Grading of mast cell tumor was performed in accordance with the Patnaik grading method [19].

**No inflammatory mammary carcinoma was included in this study.

### PD-1 is highly expressed on tumor-infiltrating lymphocytes (TILs) in canine oral melanoma

**Fig 4:**
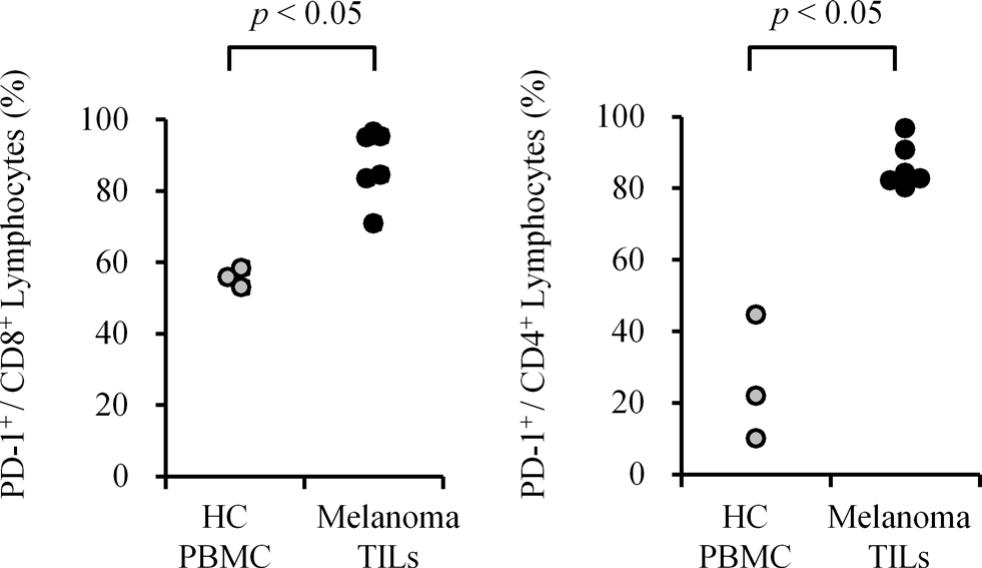
PD-1 expression on tumor-infiltrating lymphocytes (TILs) obtained from oral melanoma. TILs were collected from surgically excised oral melanoma tissues and the expression level of PD-1 was evaluated by flow cytometry. Left panel, PD-1 expression on CD8^+^ lymphocytes. Right panel, PD-1 expression on CD4^+^ lymphocytes. Peripheral blood mononuclear cells (PBMC) obtained from healthy dogs were used as control (healthy control, HC). *p* < 0.05 was considered statistically significant (Mann–Whitney U test).

In order to assess the expression level of PD-1 on tumor-associated lymphocytes, TILs were obtained from surgically excised cancer tissues, and flow cytometric analysis was conducted. Compared to peripheral blood lymphocytes from healthy dogs, PD-1 was highly expressed on both CD8^+^ and CD4^+^ TILs from oral melanoma (Fig 4, *p* < 0.05). The positive rate of PD-1 expression on CD8^+^ or CD4^+^ TILs was 70.9–96.6% or 80.2–96.8%, respectively (Table 2). In the case of hepatic tumors, more optimal control cells were available; normal tissue-infiltrating lymphocytes (NILs) from the same individuals were used. Both in hepatocellular adenoma and in hepatocellular adenocarcinoma, TILs had higher expression of PD-1 than NILs (S2 Fig), indicating that upregulation of PD-1 expression might be a common phenotype for TILs.

**Table 2:** Oral melanoma samples used in the flow cytometric analysis of PD-1 expression.

				PD-1 expression (%)	
Breed	Age	Sex	Site	CD8^+^	CD4^+^
American cocker spaniel	12	Male	Maxilla, left	84.4	84.2
Golden retriever	14	Female, spayed	Mandible, left	83.5	82.2
Miniature dachshund	13	Male	Mandible, right	70.9	80.2
Golden retriever	14	Male, castrated	Lip, upper left	95.2	82.7
Chihuahua	13	Female	Maxilla, left	95.1	96.8
Mix	9	Male	Mandible, right	96.6	90.7

Breed of dog, age, sex, tumor site, and positive rate of PD-1 expression (%) on CD8^+^ or CD4^+^ lymphocytes were shown in the table.

## Discussion

Melanoma is a relatively common type of tumor, which accounts for 7% of all malignant cancers and is the most frequent oral malignancy in dogs [20]. Canine malignant melanoma can occur in the haired skin, oral cavity, nail bed, foot pad, eye, or mucocutaneous junction [20]. Among these forms, oral melanoma is considered a highly malignant cancer because it frequently causes severe local invasion and metastatic disease. Unfortunately, oral melanoma is generally resistant to chemotherapy, making it difficult to treat metastatic lesions, which eventually kill diseased dogs [21]. The absence of effective systemic therapy encourages the development of novel therapeutic modalities, including immunotherapies. In humans, immune checkpoint inhibitors such as anti-PD-1 and anti-PD-L1 antibodies have been used for the treatment of advanced melanoma, showing promising efficacies, with the objective response rates of 28% and 18%, respectively [6,7]. In this study, immunohistochemical analysis demonstrated that as much as 90% (36/40) of dog oral melanoma expressed PD-L1, suggesting that the PD-1/PD-L1 axis is a major mechanism of immune evasion for this form of cancer. This result is well consistent with our previous report, which showed that 100% (8/8) of oral melanoma expressed PD-L1, whereas most melanoma excised from the eye or skin did not express PD-L1 [16]. Moreover, in agreement with these results, PD-1 was highly expressed on both CD8^+^ and CD4^+^ TILs obtained from oral melanoma. These TILs seemed functionally exhausted because immunosuppressive motifs, which suppress effector functions of T cells in human and mice, is highly conserved in dog PD-1 [16]. Considering that dog malignant melanoma shares some similarities with human melanoma, inhibitors of the PD-1/PD-L1 axis could also provide therapeutic effects against dog oral melanoma, and dogs could be a good animal model for investigating therapeutic aspects of PD-1/PD-L1 inhibitors against melanoma in clinical studies.

OS is the most common bone cancer in dogs, known for its highly metastatic characteristics. With amputation alone, MST for dogs with OS is only 19 weeks, and approximately 90% of the dogs develop metastatic lesions [22,23]. Therefore, aggressive surgery in combination with systemic chemotherapy has been a standard treatment for canine OS, and new systemic therapies need to be investigated. In humans, anti-PD-L1 antibodies are expected to have therapeutic effects on OS because approximately 75% (12/16) of metastatic OS showed PD-L1 expression. In a mouse model, an anti-PD-L1 antibody improved the function of cytotoxic T lymphocytes and increased the survival of tumor-bearing mice [24]. Because 70% (7/10) of primary OS in dogs were found to express PD-L1, blocking antibodies may also have a therapeutic efficacy against canine OS. However, it may be important to note that, in contrast to its metastatic lesions, no human primary OS expressed PD-L1 [24], whereas dog primary OS expressed PD-L1 at a high rate. The difference in PD-L1 expression should be taken into careful consideration when extrapolating findings from human OS studies to canine OS or vice versa.

Dogs have a relatively high risk for developing HSA [25], which is a malignant cancer originating from vascular endothelial cells. Considering that dog HSA is highly metastatic, adjuvant chemotherapy remains important although it can provide only a modest benefit in survival times [26]. Because angiosarcoma is a rare disease in humans, reports on PD-L1 expression in angiosarcoma have been limited [27,28], and its therapeutic implication has not been well studied. Because PD-L1 expression was found in 60% (6/10) of the dog HSA samples, it is worth investigating the therapeutic potential of PD-1/PD-L1 inhibitors as novel systemic immunotherapies for dog HSA. PD-L1 expression in HSA may share the characteristics of its originating vascular endothelial cells, because vascular endothelial cells express PD-L1 in response to several cytokines such as IFNs [29,30]. It may be reasonable to suggest that HSA utilizes the PD-1/PD-L1 axis as an immune evasion mechanism because PD-L1 on vascular endothelial cells has been reported to negatively regulate CD8^+^ T-cell responses such as IFN-γ production and cytolytic activity [30].

MCT is the most common cutaneous cancer in dogs, accounting for up to 21% of all canine skin neoplasms [31]. Although the clinical behavior of MCT can vary, histologic grading [19] is widely used and considered to be a reliable prognostic factor for MCT dogs. According to Bostock *et al*., for high-grade or undifferentiated MCT, MST is only 13 weeks [32] and the metastatic rate is as high as 56 to 96%. In this study, high-grade (Patnaik grade III) MCT was analyzed for PD-L1 expression, and 60% (3/5) of the samples were positively stained. This result is also well consistent with our previous report, in which 66.7% (4/6) of grade III MCTs showed PD-L1 expression [16]. Although some cytotoxic chemotherapy drugs and KIT tyrosine kinase inhibitors such as imatinib and toceranib have been used to treat MCT systemically, immune checkpoint inhibitors targeting the PD-1/PD-L1 axis also have the potential to be included in the treatment of MCT because the emergence of drug resistance is sometimes observed during chemotherapy, including that with KIT inhibitors. Although PD-L1 expression in grade I or II MCT remains to be investigated, PD-L1 expression may be related to the biological behavior of MCT because it drives CD8^+^ T-cell suppression and facilitates metastasis in mouse cancer models [33]. Further investigations are required to assess whether PD-L1 expression could be used as a prognostic marker for this complicated and heterogeneous cancer.

Mammary gland tumors are the most common neoplasms in sexually intact female dogs, accounting for 70% of all cancer cases in this subpopulation [34]. Among malignant mammary tumors, mammary adenocarcinoma represents a common histopathology, and dogs with mammary adenocarcinoma treated by surgery alone sometimes suffer from a recurrence and/or metastases. Chemotherapy has some benefits for mammary malignancies; however, in many cases it is not curative, and alternative or additional systemic therapies need to be explored. In humans, PD-L1 expression in tumor cells was observed in 34% (15/44) of patients with breast cancer, and its expression was significantly correlated with some important prognostic factors such as the expression of hormone receptors [35]. In dogs, 80% (4/5) of the mammary adenocarcinoma samples expressed PD-L1, although its association with the prognosis remains unclear. A further investigation of the relationship between the expression status of PD-L1 and the prognosis in the case of canine mammary tumors should be conducted, and it is worth to at least evaluate the therapeutic potential of PD-1/PD-L1 inhibitors as novel systemic therapies against this cancer.

Prostate adenocarcinoma is a relatively uncommon disease in male dogs. Although the incidence is low, prostate adenocarcinoma often arises as a clinical problem because local progression and metastases are the common sequelae. At the time of diagnosis, regional and distant metastases can be found in most cases, and MSTs for dogs with no treatment are considered to be less than a month [36]. To date, no consensus for the standard of care has been achieved, and conventional treatments such as surgery, radiation therapy, and chemotherapy are palliative in most dogs. In humans, 50% (8/16) of castration-resistant prostate cancer (CRPC) cases were reported to express PD-L1 [37]; however, none of the CRPC patients who received an anti-PD-1 antibody had an objective response in a clinical trial [6]. No response in the CRPC patients is possibly due to the absence of PD-L1 expression in the tumor tissues, although only a few samples from the enrolled patients were examined and found to be PD-L1-negative [6,38]. In this study, PD-L1 was expressed in 60% (3/5) of the dog prostate adenocarcinoma samples, but its therapeutic implications remain obscure. Further studies of the association of PD-L1 expression with the treatment outcome should be conducted, and dogs could be a powerful animal model for human prostate cancer because dog prostate adenocarcinoma shares some features with human prostate cancer in terms of its spontaneous occurrence, metastatic propensity, and PD-L1 expression.

The other cancers tested in this study did not show any PD-L1 expression. The absence of PD-L1 expression in these tumors may be due to differences in the origin of tumor cells, tumor site, and/or immunologic condition of each cancer. Because the sample size tested in this study was very small, further analysis should be performed before making a conclusion on the expression status of PD-L1 in those cancers. Recently, the regulatory mechanisms of PD-L1 expression in humans have been extensively investigated. One of the mechanism for inducing PD-L1 expression in tumor cells is in response to IFN-γ [9], which is likely produced by immune cells in the tumor microenvironment during antitumor immune responses. Because PD-L1 expression in canine cancer cell lines was induced or enhanced by canine IFN-γ treatment [16], this mechanism is also likely to be involved in the regulation of PD-L1 in dogs. Another mechanism is associated with oncogenic signaling; the loss of the phosphatase and tensin homolog (PTEN) function has been reported to upregulate PD-L1 expression in glioma and triple-negative breast cancer cells [39,40]. In dogs, a mutation and/or loss of PTEN were also reported in melanoma, OS, HSA, and malignant mammary gland tumors [41–44]. This may contribute, at least in part, to the aberrant PD-L1 expression in these cancers in dogs.

To the best of our knowledge, this is the first report of PD-L1 expression in various canine malignant cancers. Expression analysis of PD-L1 has important implications in a clinical application of PD-1/PD-L1 inhibitors in dogs because, in human clinical trials with an anti-PD-1 antibody, PD-L1 expression was reported to be associated with the treatment outcome. The patients with PD-L1-positive tumors had an objective response rate of 36%, while none of the patients with PD-L1-negative tumors had an objective response [6]. Our data provide basic information on the involvement of the PD-1/PD-L1 axis in canine cancer immunology and strongly encourage the development of novel immunotherapies to target the PD-1/PD-L1 pathway in dogs. Because anti-PD-L1 antibodies were shown to have therapeutic potential for canine cancers [16], clinical applications of anti-PD-L1 antibodies deserve further investigation.

## Supporting Information

S1 Fig Representative immunohistochemistry with isotype-matched control antibody. Oral melanoma specimen was stained with rat IgM isotype control antibody. No staining was observed. Original magnification, 200×.
(TIF)

S2 Fig PD-1 expression on TILs obtained from hepatic tumors. TILs obtained from (A) hepatocellular adenoma and (B) hepatocellular adenocarcinoma were analyzed for PD-1 expression by flow cytometry (red line). Normal tissue-infiltrating lymphocytes (NILs) obtained from adjacent healthy liver tissue of the same individuals (blue line) were used as control. Black line, isotype control for TILs. Shaded area, isotype control for NILs.
(TIF)
