# Supplementary figures and images for: Immunohistochemical Analysis of PD-L1 Expression in Canine Malignant Cancers and PD-1 Expression on Lymphocytes in Canine Oral Melanoma
Source: PLoS One. 2016 Jun 8;11(6):e0157176. doi: 10.1371/journal.pone.0157176 (PMC4898770; doi:10.1371/journal.pone.0157176)

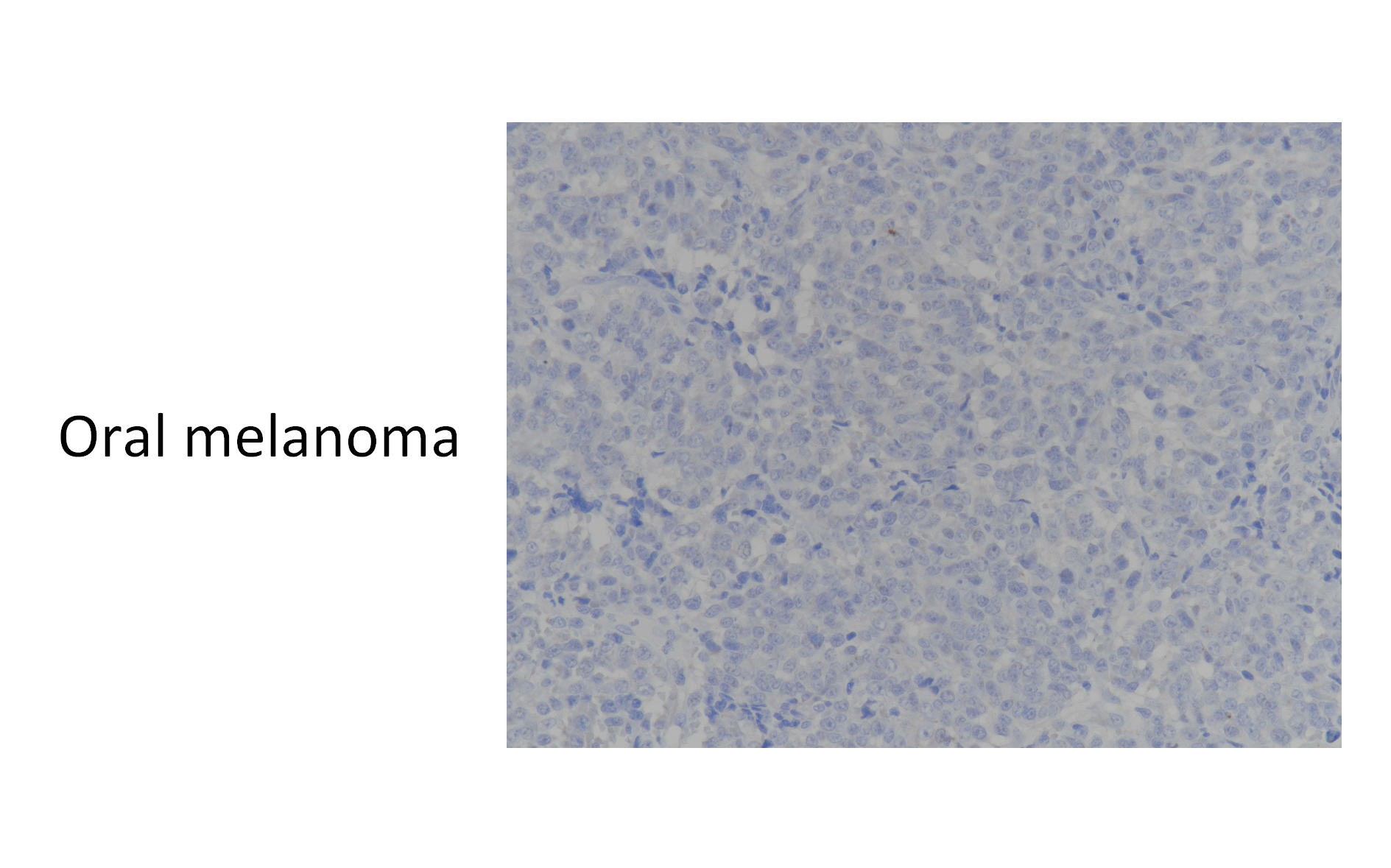

Supplement: S1 Fig — Oral melanoma specimen was stained with rat IgM isotype control antibody. No staining was observed. Original magnification, 200×. (TIF) [file pone.0157176.s001.tif]

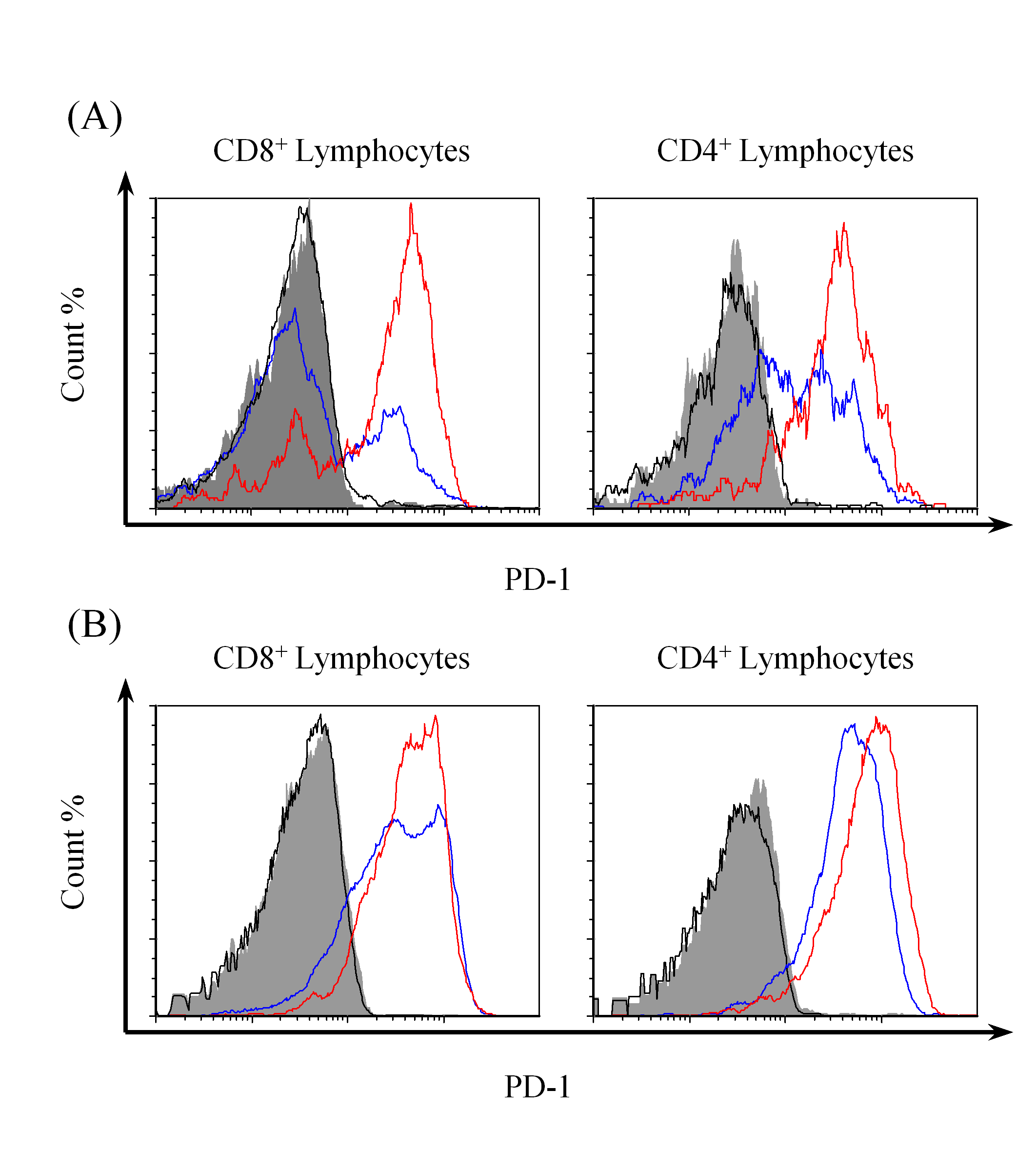

Supplement: S2 Fig — TILs obtained from (A) hepatocellular adenoma and (B) hepatocellular adenocarcinoma were analyzed for PD-1 expression by flow cytometry (red line). Normal tissue-infiltrating lymphocytes (NILs) obtained from adjacent healthy liver tissue of the same individuals (blue line) were used as control. Black line, isotype control for TILs. Shaded area, isotype control for NILs. (TIF) [file pone.0157176.s002.tif]
